# Temperature Effects on Methanogenesis and Sulfidogenesis during Anaerobic Digestion of Sulfur-Rich Macroalgal Biomass in Sequencing Batch Reactors

**DOI:** 10.3390/microorganisms7120682

**Published:** 2019-12-11

**Authors:** Heejung Jung, Jaai Kim, Changsoo Lee

**Affiliations:** School of Urban and Environmental Engineering, Ulsan National Institute of Science and Technology (UNIST), Ulsan 44919, Korea; hjjung1018@unist.ac.kr (H.J.); jaai@unist.ac.kr (J.K.)

**Keywords:** anaerobic digestion, mesophilic, methanogenesis, sulfidogenesis, thermophilic

## Abstract

Methanogenesis and sulfidogenesis, the major microbial reduction reactions occurring in the anaerobic digestion (AD) process, compete for common substrates. Therefore, the balance between methanogenic and sulfidogenic activities is important for efficient biogas production. In this study, changes in methanogenic and sulfidogenic performances in response to changes in organic loading rate (OLR) were examined in two digesters treating sulfur-rich macroalgal waste under mesophilic and thermophilic conditions, respectively. Both methanogenesis and sulfidogenesis were largely suppressed under thermophilic relative to mesophilic conditions, regardless of OLR. However, the suppressive effect was even more significant for sulfidogenesis, which may suggest an option for H_2_S control. The reactor microbial communities developed totally differently according to reactor temperature, with the abundance of both methanogens and sulfate-reducing bacteria being significantly higher under mesophilic conditions. In both reactors, sulfidogenic activity increased with increasing OLR. The findings of this study help to understand how temperature affects sulfidogenesis and methanogenesis during AD.

## 1. Introduction

Anaerobic digestion (AD) is widely applied in the treatment of various organic wastes or wastewaters, and biogas (mainly CH_4_ and CO_2_) produced from AD is considered a promising renewable energy source. Sulfate is a common constituents of various wastes or wastewaters, with varying concentrations of 50–200 mg/L in municipal sewage and even as high as 50 g/L in wastewaters from the pulp and food industries [1,2]. Sulfate is reduced to hydrogen sulfide (H_2_S) via dissimilatory sulfate reduction by sulfate-reducing bacteria (SRBs) in anaerobic environments, which is a major concern in AD [3]. H_2_S is toxic to anaerobic microorganisms including methanogens and can cause deterioration of methanogenesis. In addition, the odorous and corrosive properties of H_2_S can bring about hygiene issues and equipment damage, respectively. In AD systems, methanogens and SRBs compete for common substrates (i.e., hydrogen and acetate), and the outcome of competition is largely determined by the reaction thermodynamics and growth kinetics [4]. Generally, SRBs have competitive advantages over methanogens owing to their higher growth rate and substrate affinity [5,6]. Furthermore, sulfidogenesis from hydrogen and acetate is thermodynamically more favorable than their methanogenesis [7]. Accordingly, sulfidogenesis often outcompetes methanogenesis in sulfur-rich environments. However, various environmental factors, such as temperature, pH, sulfate concentration, and microbial acclimation, can complicate the competitive interaction between methanogens and SRBs [8,9].

Temperature is a major environmental factor controlling microbial community structure and activity in AD systems [10]. Although methanogenesis and sulfidogenesis occur at both mesophilic and thermophilic temperatures [4,8,11,12], divergent results have been observed on their competition under the different temperature conditions (Table S1). Highly comparable electron flows to methanogenesis and sulfidogenesis were observed in a down-flow digester treating a synthetic mixture of acid mine drainage and vinasse at mesophilic temperature [13]. Mesophilic studies in up-flow anaerobic sludge blanket (UASB) reactors reported that methanogenesis was more competitive when treating a mixture of acetate and ethanol [4,14], while sulfidogenesis is predominant when treating methanol [15] or ethanol [16]. The observations on the competition between methanogenesis and sulfidogenesis at thermophilic temperature also varied. Methanogenesis predominated in mesophilic UASB reactors treating methanol [4,11] or formate [17]. Meanwhile, the predominance of sulfidogenesis was observed in UASB reactors fed with methanol [17] or a mixture of acetate, propionate, and butyrate [18].

Such conflicting observations can be attributed to the use of different substrates, such as methanol, ethanol, formate, or acetate, for which methanogens and SRBs may have different preferences [17]. Tests using synthetic media with simple substrate organics as above have limitations in representing real AD plants treating waste streams with complex composition. In addition, most previous studies examined methanogenesis and sulfidogenesis at either mesophilic or thermophilic temperatures or with a stepwise transition of temperature, rather than comparing at different temperatures in parallel. Given the intricate interactions between methanogens and SRBs and the competitive effect of sulfidogenesis on methanogenesis, it is worthwhile to comparatively investigate their behavior and interactions under different temperature conditions. However, so far, limited information is available on whether and how methanogens and SRBs respond and compete differently during AD at different temperatures. To address this gap, this study aimed to (1) examine the effects of temperature on methanogenesis and sulfidogenesis, and on their relationship, during the AD of sulfur-rich macroalgal biomass, at mesophilic and thermophilic temperatures in parallel and (2) compare the responses of methanogenic and sulfidogenic activities to increasing organic loads under the different temperature conditions. To gain a more comprehensive insight, microbial community structure and dynamics were analyzed based on the concentrations of not only bacterial and archaeal 16S rRNA genes but also a functional gene specific to SRBs (*dsrA*), and the results were linked to performance data. The findings of this study will help to better understand the competition between methanogenesis and sulfidogenesis at mesophilic and thermophilic temperatures and will be a useful addition to the literature on the AD of sulfur-rich feedstocks.

## 2. Materials and Methods

### 2.1. Inoculum and Substrate

Anaerobic sludge taken from a full-scale AD plant co-digesting food waste and sewage sludge was used as inoculum. Fresh *Ulva* biomass was collected from a local beach (Busan, Korea), rinsed with a small amount of tap water to remove impurities, and ground to a slurry in a kitchen blender. The *Ulva* slurry was diluted to obtain a chemical oxygen demand (COD) concentration of 5–20 g/L according to the designed organic loading rates (OLRs). The basic physicochemical characteristics of the inoculum and substrate used are determined (Table S2).

### 2.2. Reactor Operation

**Figure 1:**
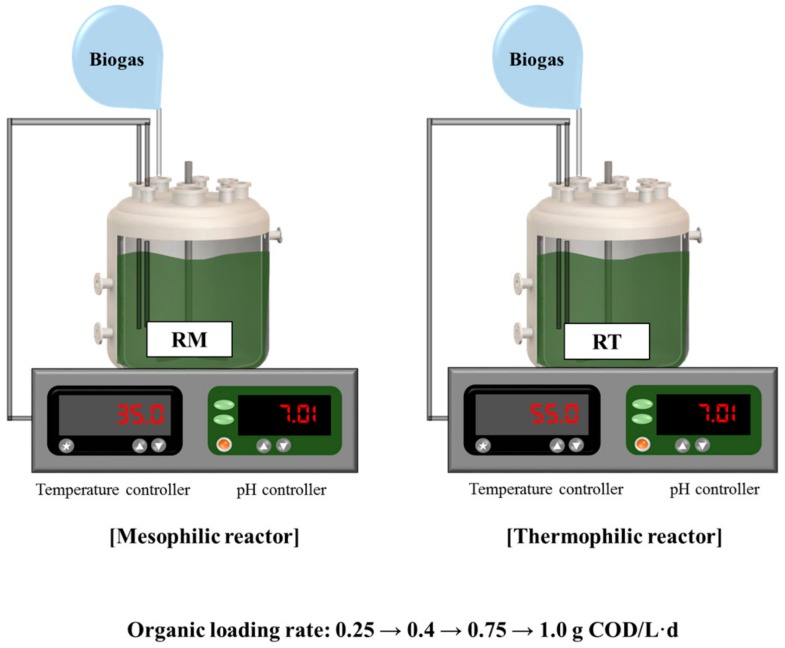
Schematic diagram of the experimental setup.

Two anaerobic reactors with a working volume of 2 L, named RM and RT, were operated in sequencing batch mode at mesophilic (35 ± 2 °C) and thermophilic (55 ± 2 °C) temperatures, respectively (Figure 1). The reactors were initially filled with inoculum (100%, *v*/*v*) and purged with N_2_ for 20 min to remove residual oxygen before feeding. Both reactors were maintained at neutral pH by adding 3 N NaOH solution during operation. One sequencing batch cycle consisted of four stages in sequence: filling (<10 min), reacting (21 h), settling (3 h), and drawing (<10 min). The reactors were tested with increasing OLR (0.25, 0.4, 0.75, and 1.0 g COD/L·d) by increasing the substrate concentration at a constant hydraulic retention time of 20 days. Steady-state performance data at each OLR were obtained after at least three turnovers of the reactor working volume.

### 2.3. Physicochemical Analysis

COD concentration was determined spectrophotometrically using an HS-COD-MR kit (HUMAS). The concentrations of volatile fatty acids (VFAs, C_2_–C_7_) were measured using an Agilent 7820A FID gas chromatograph equipped with an Innowax column (Agilent, Santa Clara, CA, USA). Samples for soluble COD and VFA measurements were prepared by filtration through a 0.45-µm pore-size syringe filter. Total dissolved sulfide (TDS) was quantified spectrophotometrically using an HS-S kit (HUMAS). The contents of CH_4_, CO_2_, and H_2_ in biogas were measured using an Agilent 7820A TCD gas chromatograph coupled with a ShinCarbon ST column (Restek, Belfont, PA, USA), and the H_2_S content was determined using gas detector tubes (4H and 4HH, GASTEC). Produced biogas volume in each reactor was measured by water displacement and corrected to standard temperature and pressure (0 °C and 1 bar). Solids were measured according to the procedures in Standards Methods [19]. Elemental (C, H, O, N, and S) contents of inoculum and substrate were determined on a dry weight basis using an organic elemental analyzer (Flash 2000, Thermo Scientific, Waltham, MA, USA). All analyses described above were carried out at least in duplicate.

### 2.4. Real-Time Polymerase Chain Reaction

Mixed liquor samples for microbial community analysis were collected from the reactors during steady-state operation at each OLR applied. Total DNA was extracted from the reactor biomass samples using an automated nucleic acid extractor (Exiprogen, Bioneer, Daejeon, Korea) according to the manufacturer’s instructions. A 1-mL mixed liquor was pelleted at 13,000 g for 3 min and repeatedly washed by resuspending in distilled water (up to 1 mL), discarding the supernatant (900 µL), and pelleting (13,000 g for 3 min). A 200-μL aliquot of the final resuspension was loaded onto the automated extractor with the ExiProgen Bacteria Genomic DNA kit (Bioneer). The purified DNA was recovered in 100 μL of elution buffer and stored at −20 °C until use.

Real-time polymerase chain reaction (PCR) was employed to estimate the abundance of target microbial groups, i.e., bacteria, methanogens, and SRBs. Five 16S rRNA gene-specific primers and probe sets targeting the domain Bacteria and four methanogenic groups (i.e., the orders *Methanobacteriales* and *Methanomicrobiales* and the families *Methanosarcinaceae* and *Methanotrichaceae* (formerly *Methanosaetaceae*)), respectively, were used as previously described [20]. For SRB quantification, real-time PCR was performed with a primer set specific for the dissimilatory sulfite reductase gene (*dsrA*), a molecular marker for SRBs, as previously described [21]. The amplification mixtures for bacterial and methanogenic 16S rRNA genes were prepared with THUNDERBIRD Probe qPCR Mix (TOYOBO, Osaka, Japan), and those for *dsrA* were with THUNDERBIRD SYBR qPCR Mix (TOYOBO), as described previously [22]. PCR amplification with fluorescence monitoring was carried out in a QuantStudio 12K Flex system (Life Technologies, Carlsbad, CA, USA). The concentration of a target sequence in unknown samples was determined against the corresponding standard curve prepared as described previously [22]. Each sample was analyzed in duplicate.

### 2.5. Cluster Analysis

A quantitative matrix was generated based on the relative abundance of each target group in the total methanogenic population, on the basis of 16S rRNA gene concentration measured by real-time PCR. The obtained matrix was subjected to cluster analysis with the unweighted pair group method with arithmetic means (UPGMA) algorithm. The calculations to construct a dendrogram were performed based on the Sorensen distance measure using PAST (ver. 3.25) (http://folk.uio.no/ohammer/past/).

## 3. Results and Discussion

### 3.1. Methanogenic Performance

**Figure 2:**
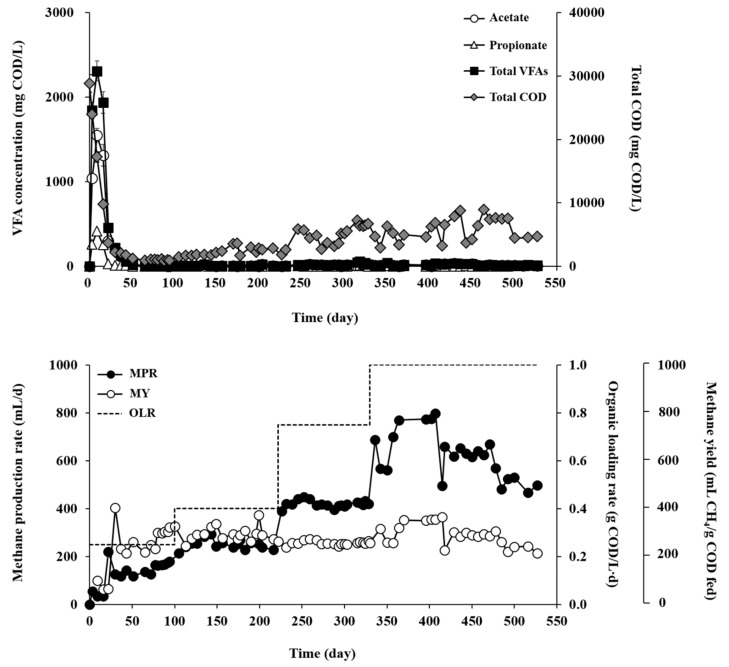
Organic removal and methane production profiles in RM (mesophilic).

**Figure 3:**
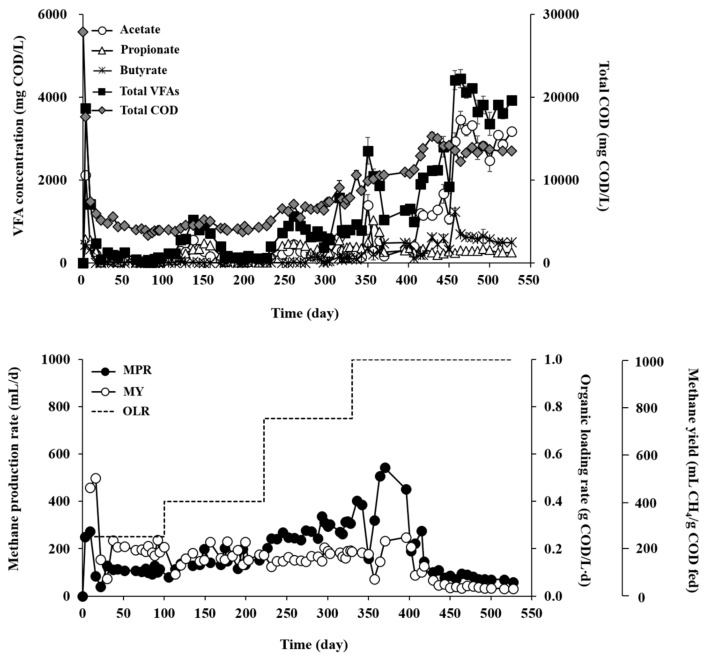
Organic removal and methane production profiles in RT (thermophilic).

RM and RT showed very different AD performances during the experiment for over 500 days at increasing OLRs (Figure 2 and Figure 3). RM maintained significantly better and more stable performance, in terms of organic removal, residual VFAs, and methane production, than RT at all tested OLRs (Table 1). The COD removal efficiency ranged from 56.6% to 81.9% across the experimental phases with different OLRs in RM, while it ranged only from 21.2% to 49.9% in RM. The observation of the lowest COD removal efficiency at an OLR of 0.25 g COD/L·d in RT may be attributed to temperature stress on the inoculum microorganisms collected from a mesophilic digester [23]. Microorganisms that are not thermotolerant may have been damaged in RT by exposure to thermophilic temperature, which can result in cell death and release of cell debris and thus adversely affect the treatment performance and effluent quality. In support of this, the effluent concentration of volatile suspended solids (VSS) was 2.1-fold higher in RT than in RM effluent at OLR of 0.25 g COD/L·d (data not shown), presumably owing to the discharge of inactive or dead microorganisms and cell debris with the decanted supernatant [24]. Although the COD removal efficiency of RT increased somewhat, as microorganisms gradually acclimated to the reactor temperature, with increasing OLR, it still remained in much lower ranges than those of RM.

VFAs (mostly acetate and propionate) rapidly accumulated up to 2309 mg COD/L during the initial period of operation in RM (Figure 2). The reactor recovered rapidly from the transient imbalance between acetogenesis and methanogenesis. The residual VFA levels dropped along with increased methane production and remained stable below 50 mg COD/L until the end of the reactor operation. In RT, acetate, propionate, and butyrate were the main VFA species (Figure 3). Unlike RM, RT showed great fluctuations in VFA profiles throughout the experiment with increasing OLR. At an OLR of 1.0 g COD/L·d, the total VFA concentration reached the range of 3352–4446 mg COD/L, indicating a significant process imbalance, i.e., slow methanogenic conversion of acidogenic products, particularly acetate. This result suggests that both acetate-utilizing methanogens and SRBs were in an unfavorable environment for their activity in RT. VFA accumulation during AD under thermophilic conditions, particularly at high OLRs, has been commonly observed in digesters inoculated with mesophilic biomass [25,26].

Corresponding to the organic removal data, methanogenic performance was significantly higher, in terms of rate and yield, in RM than in RT. As the OLR increased from 0.25 to 1.0 g COD/L·d, the methane production rate (MPR) of RM gradually increased from 86.5 to 249.0 mL/L·d, whereas the methane yield (MY; per substrate COD fed) decreased from 320 to 230 mL/g COD fed (Table 1). Nevertheless, the MY values are still reasonable and comparable to those previously reported in mesophilic digesters treating sulfur-rich feedstocks including *Ulva* biomass [21,27,28], and MY often tends to decrease with increasing OLR [29,30,31]. On the other hand, the MPR of RT gradually increased from 57.3 to 156.0 mL/L·d with increasing OLR from 0.25 to 0.75 g COD/L·d, but a further increase in OLR to 1.0 g COD/L·d caused a significant drop in MPR to 33.1 mL/L·d. The MY of RT dropped significantly from 210 mL/g COD fed at an OLR of 0.25 g COD/L·d to 30 mL/g COD fed at an OLR of 1.0 g COD/L·d. These results imply that 1.0 g COD/L·d was the critical OLR for RT at which the methanogenic activity was retarded significantly, thereby leading to serious process imbalance. Thermophilic AD of *Ulva* biomass as the sole substrate has rarely been studied in continuous mode, and little literature is available for comparison. However, the MY values obtained in RT, except for that at the highest OLR of 1.0 g COD/L·d, are higher than those reported for batch thermophilic AD of *Ulva* biomass [32,33] Overall, RT was more susceptible to OLR increase than RM, particularly at higher OLRs.

### 3.2. Sulfidogenic Performance

**Figure 4:**
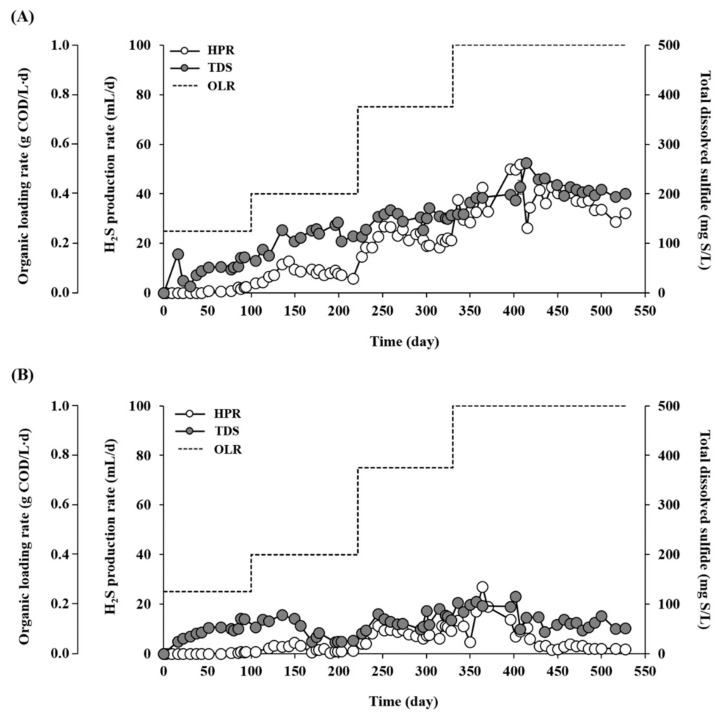
H_2_S production rate (HPR) and total dissolved sulfide (TDS) concentration in RM (mesophilic, (**A**)) and RT (thermophilic, (**B**)).

Sulfidogenesis was also much more active in RM than in RT throughout the experiment (Figure 4). A measurable content of H_2_S was first detected in the biogas after three turnovers of the working volume in RT, whereas it was detected after only one turnover in RM. The H_2_S production rate (HPR) and effluent TDS concentration in RM increased from 1.17 to 15.78 mL/L·d and from 65.7 to 201.1 mg S/L, respectively, with increasing OLR from 0.25 to 1.0 g COD/L·d (Table 1). Correspondingly, the H_2_S yield (HY, per substrate COD fed) of RM also increased from 4 to 14 mL H_2_S/g COD fed with the increase in OLR. Although significantly lower than that of RM, the HPR of RT gradually increased up to 5.31 mL/L·d with increasing OLR until 0.75 g COD/L·d. However, as observed for methanogenic performance, it dropped suddenly to 1.0 mL/L·d with a further increase in OLR to 1.0 g COD/L·d. Compared to RM, RT showed much lower HY (0.9–6.5 mL H_2_S/g COD fed) and TDS concentration (25.3–73.7 mg S/L) in the tested OLR range.

The H_2_S content in biogas was high throughout the experiment in both reactors, although it was substantially lower in RT (4000–19,500 ppmv) than in RM (9233–37,000 ppmv). These values greatly exceed the limits for use of biogas, for example, <10 ppmv for kitchen stoves, <500 ppmv for combustion engines, and <1000 ppmv for boilers [34]. Therefore, a purification step for H_2_S removal is necessary for the biogas produced from both RM and RT for its use as an energy source. Given that H_2_S removal is a costly post-treatment process, this result suggests that finding an effective way to control sulfide formation is a key to improve the feasibility and economy of the biomethanation of sulfur-rich feedstocks.

### 3.3. Temperature Effects on Methanogenesis and Sulfidogenesis

**Figure 5:**
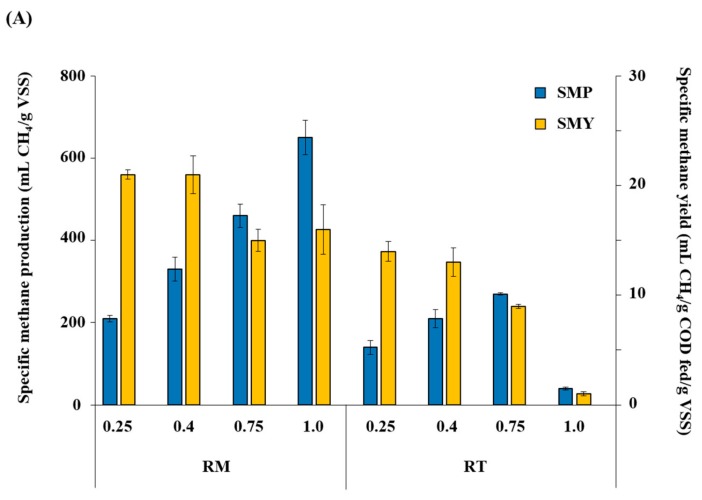

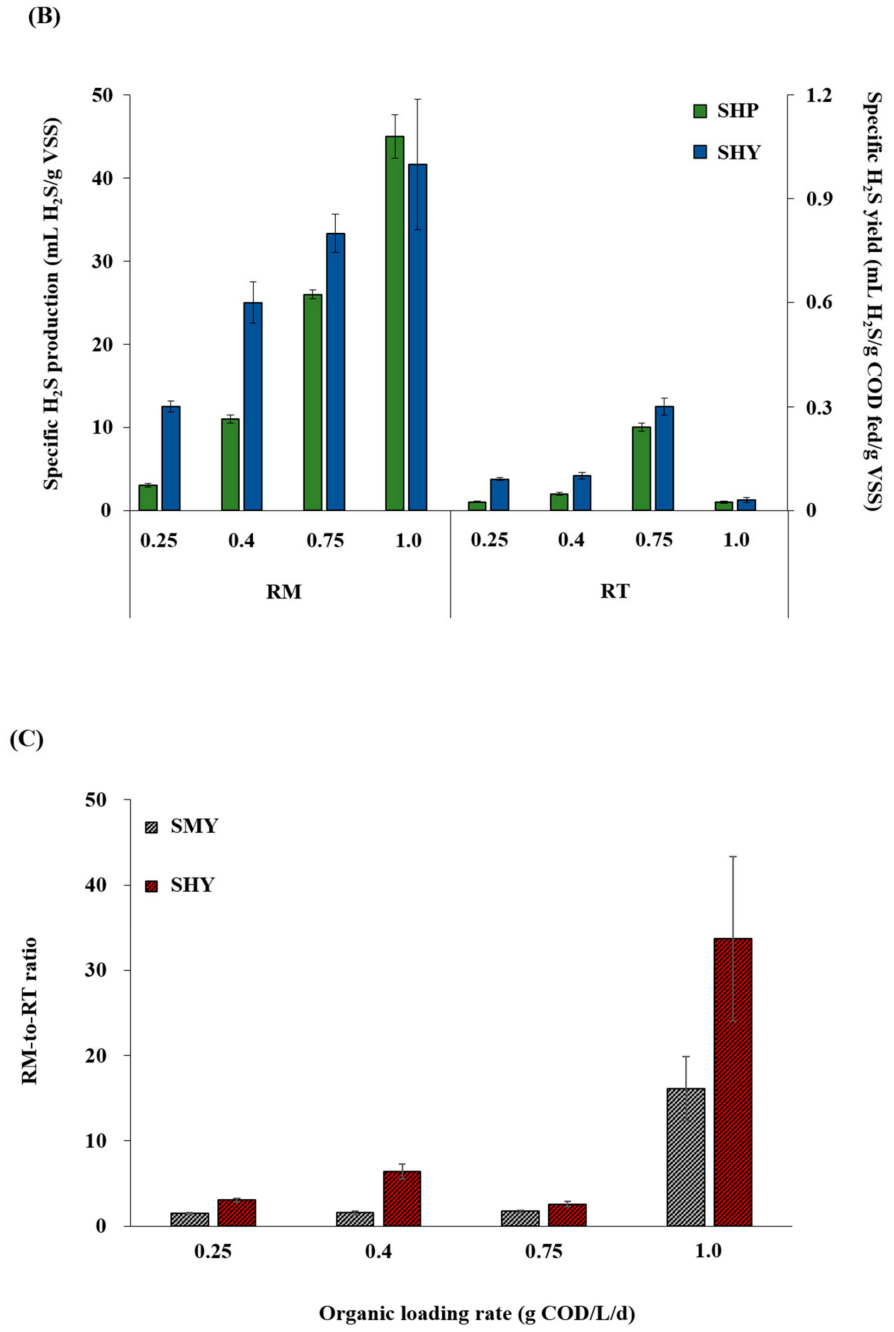
Changes in specific methane production (SMP) and yield SMY; (**A**) and specific H_2_S production (SHP) and yield SHY; (**B**) with increasing organic loading rate (OLR) in RM (mesophilic) and RT (thermophilic), and the ratios of SMY and SHY in RM to those in RT (RM-to-RT ratio) at each OLR (**C**). Samples are labeled with the corresponding reactor names and OLRs (days).

The reactor operating temperature strongly affected both methanogenic and sulfidogenic activities. Specific methane production (SMP) increased relative to the increase in OLR to reach the maximum value of 650 mL/g VSS at an OLR of 1.0 g COD/L·d in RM (Figure 5A). This result shows that RM maintained stable methanogenic activity balanced with acidogenic activity at all tested OLRs. In contrast, the SMP in RT increased until the OLR reached 0.75 g COD/L·d to show the highest value of 270 mL/g VSS, but decreased sharply to 40 mL/g VSS along with a sudden buildup of VFAs when the OLR was further increased to 1.0 g COD/L·d (Figure 3 and Figure 5A). This result confirms again that 1.0 g COD/L·d was the critical OLR for RT, which caused a serious imbalance between acidogenesis and methanogenesis. Interestingly, in contrast to SMP, specific methane yield (SMY) showed a tendency to decease with increasing OLR in both reactors, particularly in RT. The SMY of RT decreased drastically from 14 mL/g COD fed/g VSS at an OLR of 0.25 g COD/L·d to 1 mL/g COD fed/g VSS at an OLR of 1.0 g COD/L·d, while that of RM remained between 15 and 21 mL/g COD fed/g VSS with small changes during the experiment. This observation indicates that the methanogenic community in RT was more susceptible to OLR increase, leading to a serious process imbalance at higher OLRs.

The methane content in biogas decreased with increasing OLR in both reactors (Table 1), meaning that methanogenesis was diminished by increased organic load. Similar observations of methane content reduction with increasing OLR, particularly under overloading conditions, have been reported in previous studies [35,36]. It is notable that the decrease in methane content was far more significant in RT than in RM, particularly at an OLR of 1.0 g COD/L·d. Such differences in methane content and in its variations with increasing OLR can be attributed to the different methanogenic activities of the RM and RT microbial communities. It is also worth noting that the methane content is affected by the changes in gas solubility in water according to temperature (mole fraction in liquid phase): 4.8 × 10^−4^ at 35 °C and 3.4 × 10^−4^ at 55 °C for CO_2_ and 2.2 × 10^−5^ at 35 °C and 1.8 × 10^−5^ at 55 °C for CH_4_ [37].

Both specific H_2_S production (SHP) and specific H_2_S yield (SHY) increased with increasing OLR and reached the highest values at an OLR of 1.0 g COD/L·d in RM (Figure 5B), indicating that the increased organic load provided favorable conditions for sulfidogenesis. One thing to note is that, unlike SMY (Figure 5A), SHY increased greatly from 0.30 mL/g COD fed/g VSS at an OLR of 0.25 g COD/L·d to 1.0 mL/g COD fed/g VSS at an OLR of 1.0 g COD/L·d. This result suggests that the sulfidogenic activity per unit biomass could likely further increase and handle greater food-to-microorganism ratios. In RT, SHA and SHY increased with increasing OLR until 0.75 g COD/L·d; however, their values and the extent of their increase were much lower than those in RM. In addition, a further increase in OLR to 1.0 g COD/L·d resulted in a sharp decrease in both SHA and SHY (>10-fold decrease relative to those at 0.75 g COD/L·d). This indicates that 1.0 g COD/L·d was the critical OLR, which had a significant adverse effect not only on methanogenesis but also on sulfidogenesis.

Therefore, it is concluded that both methanogenic and sulfidogenic activities were lower, or substantially suppressed, in RT relative to RM throughout the experiment. This is also well reflected in the RM-to-RT ratios of SMY and SHY, which were significantly greater than 1 at all tested OLRs (Figure 5C). The ratios were both markedly higher at an OLR of 1.0 g COD/L·d than at any other OLR applied, reflecting the severe process imbalance observed in RT at this OLR (Figure 3 and Table 1). An important point to note is that the RM-to-RT ratio of SHY (2.6–33.7) was consistently higher than that of SMY (1.5–16.1) at all tested OLRs. This result may suggest that the extent of suppression by the thermophilic temperature (55 ± 2 °C) was greater for sulfidogenesis than for methanogenesis, although both were less active in RT than in RM.

### 3.4. Methanogen Community Structures

**Figure 6:**
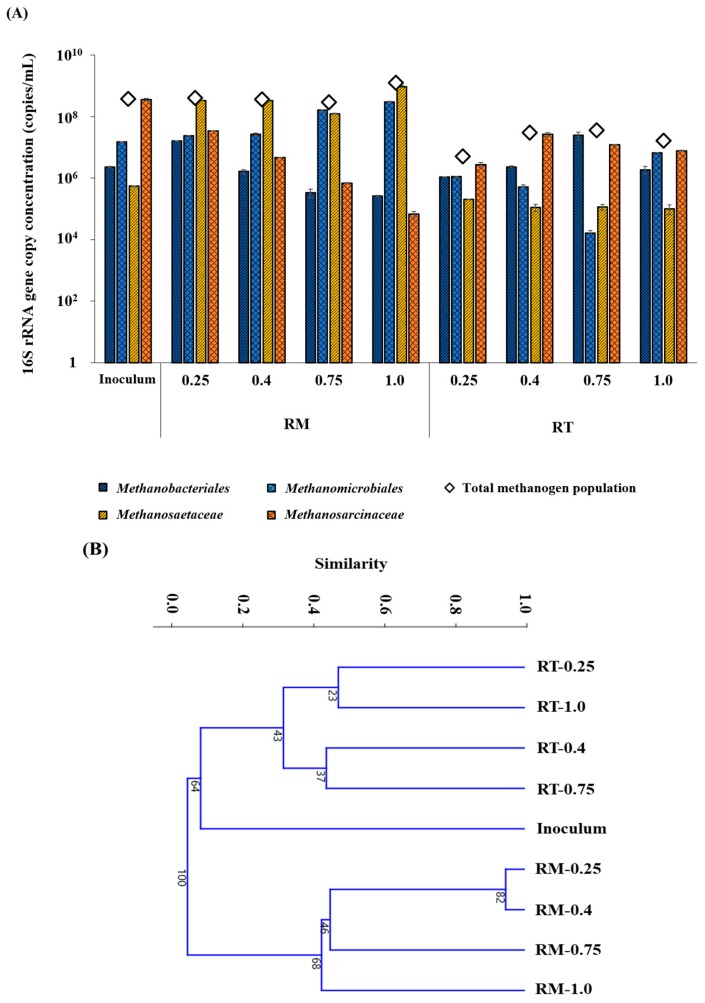
Changes in methanogenic 16S rRNA gene concentrations with increasing OLR in the experimental reactors (**A**) and cluster dendrogram generated based on the relative abundances of target methanogenic groups (**B**). Samples are labeled with the corresponding reactor names (RM, mesophilic; RT, thermophilic) and OLRs (days).

In RM, *Methanotrichaceae* was the major methanogenic group (42.9%–91.0%), indicating that aceticlastic methanogenesis was likely the major pathway for methane production (Figure 6A). *Methanotrichaceae* is generally considered indicative of a balanced AD with low residual VFAs [38]. The methanogenic community structures of RM and inoculum, in which *Methanosarcinaceae* was predominant, were significantly different, although both originated from mesophilic systems (Figure 6A). This difference can be attributed to the different substrate characteristics, which significantly affects the development of microbial community structure in AD processes. Hydrogenotrophic methanogens, particularly *Methanomicrobiales*, became more abundant with increasing OLR in RM. This result suggests that the contribution of hydrogenotrophic methanogenesis to the methane production from RM increased at higher OLRs. In contrast to *Methanomicrobiales*, the *Methanobacteriales* concentration decreased as the OLR increased. A possible explanation for the difference between these hydrogenotrophic groups is that *Methanobacteriales* is more sensitive than *Methanomicrobiales* to H_2_S toxicity [39] and organic overload [40]. Dominance shifts between *Methanobacteriales* and *Methanomicrobiales* have previously been observed with increasing OLR in digesters treating olive mill wastewater [40] and *Ulva* biomass [21].

The total methanogen population (TMP; sum of the 16S rRNA gene concentrations of all target methanogenic groups) remained considerably lower (7.9–78.9-fold) in RT than in RM at all tested OLRs (Figure 6), corresponding to the significantly lower methane productivity of RT than that of RM throughout the experiment (Table 1). The lower TMP in RT was possibly due to the damage to mesophilic microorganisms caused by the thermophilic temperature, as discussed above. The abundance and diversity of methanogens are often higher in mesophilic than in thermophilic digesters [41,42]. In RT, *Methanobacteriales* and *Methanomicrobiales* accounted for 7.8%–66.8% and 0.1%–40.6% of TMP, respectively, during the experiment. This high relative abundance of hydrogenotrophic methanogens accords with the finding that hydrogenotrophic methanogenesis coupled with syntrophic acetate oxidation is often favored over aceticlastic methanogenesis under thermophilic conditions [43]. Between two aceticlastic methanogenic families, *Methanosarcinaceae* (32.8%–90.1% of TMP) consistently outcompeted *Methanotrichaceae* (0.3%–4.0% of TMP) in RT throughout the experiment, which contrasts with the observation in RM. Fast-growing *Methanosarcinaceae* generally prevails over slow-growing *Methanotrichaceae* at high residual VFA concentrations [44]. Therefore, the dominance of *Methanosarcinaceae* over *Methanotrichaceae* appears to be related to the relatively high levels of residual VFAs (i.e., process imbalance) in RT. The dominance shifts to hydrogenotrophic methanogens with increasing OLR, within the range of 0.25–0.75 g COD/L·d before the severe performance deterioration at 1.0 g COD/L·d (Table 1), suggests that hydrogenotrophic pathway became the main route of methanogenesis at higher OLRs. This observation may be related to the higher resistance of hydrogenotrophic methanogens to environmental stress, given that the toxicity of unionized H_2_S increases with temperature [9].

In the cluster dendrogram, the methanogenic community structures of RM and RT were grouped into two separate clusters distantly related to each other and to that of the inoculum (Figure 6B). This result confirms that the methanogenic communities of RM and RT developed from the inoculum community in very different ways during the experiment. The divergence between the RM and RT methanogenic community structures appears to be characterized by the difference in dominance relationships within the hydrogenotrophic and aceticlastic groups (Figure 6A) The methanogenic community structure changed significantly with increasing OLR in both reactors, although the community structures from the same reactor were more closely related to each other than to those from the other reactor. These results suggest that operating temperature had the dominant effect on the development of methanogenic communities, and therefore the process performance, while OLR had a weaker but still significant effect in the tested OLR range.

### 3.5. Quantitiative Dynamics of Methanogens and SRBs

**Figure 7:**
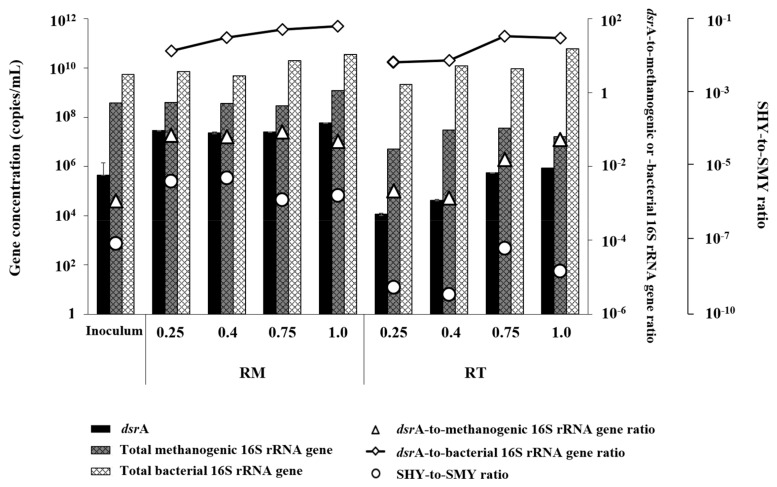
Changes in the abundances of sulfate-reducing bacteria, total methanogens, and total bacteria with increasing OLR in the experimental reactors. The specific H_2_S yield-to-specific methane yield ratio (SHY-to-SMY ratio) for each sample is shown. Samples are labeled with the corresponding reactor names (RM: mesophilic; RT, thermophilic) and OLRs (days).

Both methanogens, measured as TMP, and SRBs, measured as the concentration of *dsrA*, remained significantly higher in RM than in RT at all tested OLRs: 7.9–78.9-fold higher TMP and 44.3–2518.8-fold higher *dsrA* concentration (Figure 7). This result corresponds to the lower methanogenic and sulfidogenic activities in RT than in RM throughout the experiment (Table 1). Interestingly, the total bacterial population, measured as the concentration of bacterial 16S rRNA gene, in RT was comparable to that in RM, while both methanogens and SRBs existed in significantly lower amounts in RT. This observation supports that acidogenesis was active in both reactors and the serious process imbalance in RT was due to the slow consumption of acidogenic products by methanogenesis or sulfidogenesis. It is notable that the concentrations of methanogens and SRBs in RT were even lower than those in the inoculum, which confirms again that both methanogenic and sulfidogenic activities were suppressed at the thermophilic temperature applied.

Interestingly, the SHY-to-SMY ratio tended to rise with increasing OLR in both reactors, although it remained lower in RT than in RM throughout the experiment. This result indicates that an increasing proportion of electrons flowed to H_2_S (i.e., changes in metabolic properties of microbial community) at higher OLRs in both reactors. It appears that the activity of SRBs was promoted by increased organic load under both temperature conditions. It is worth noting that the abundance of SRBs relative to total methanogens (i.e., the *dsrA*-to-methanogenic 16S rRNA gene ratio) and the abundance of SRBs to total bacteria (i.e., the *dsrA*-to-bacterial 16S rRNA gene ratio) were both consistently lower in RT than in RM during the experiment. This result further supports that sulfidogenic activity was more significantly suppressed relative to methanogenic activity under the thermophilic conditions in RT, although both were adversely affected under thermophilic conditions. Therefore, thermophilic operation may provide a way to effectively suppress sulfidogenesis, with relatively less effect on methanogenesis.

For practical application, the adverse effect of thermophilic operation on methanogenesis and energy balance should be minimized, for example, by optimizing the reactor temperature, isolating or enriching inoculum consortia, and bioaugmenting with thermophilic methanogens. In addition, it should be considered that the inoculum was from a mesophilic digester for both RM and RT, which adversely affected the development of microbial community structure and activity during the thermophilic operation, particularly during the initial period, in RT. This is a point that deserves more detailed investigation, given that most full-scale AD plants are run under mesophilic conditions and thermophilic digesters are often inoculated with mesophilic anaerobic sludge. H_2_S generation is an inevitable and costly problem to manage in AD plants, especially when treating sulfur-rich feedstocks, and the findings of this study warrant further research.

## 4. Conclusions

Methanogenic and sulfidogenic responses to OLR changes were comparatively examined between two anaerobic reactors treating sulfur-rich macroalgal biomass under mesophilic and thermophilic conditions. The operating temperature had a decisive effect on the development of microbial communities, and therefore the methanogenic and sulfidogenic activities, during the AD in the experimental reactors. The contribution of hydrogenotrophic methanogenesis to methane production tended to increase with increasing OLR, and so did sulfidogenic activity, in both reactors. Although both methanogenesis and sulfidogenesis were markedly reduced at thermophilic temperature, the suppressive effect was significantly stronger for sulfidogenesis, which may suggest an option for in-situ sulfide control.

## Figures and Tables

**Table 1:** Steady-state performance data of the experimental reactors at increasing organic loading rates.

		RM (Mesophilic)				RT (Thermophilic)			
OLR	g COD/L·d	0.25	0.4	0.75	1.0	0.25	0.4	0.75	1.0
COD removal	%	81.9 (1.2)	65.3 (2.1)	56.6 (1.1)	77.2 (0.7)	21.2 (0.9)	46.9 (2.5)	49.9 (2.6)	32.2 (0.5)
CH_4_ content	%	68.3 (0.9) ^a^	63.8 (1.2)	59.2 (0.4)	58.4 (1.6)	73.3 (1.1)	67.3 (1.2)	57.2 (1.0)	49.5 (0.8)
MPR ^b^	mL/L·d	86.5 (3.2)	119.9 (6.2)	211.3 (4.2)	249.0 (15.6)	57.3 (7.0)	73.1 (5.4)	156.0 (2.0)	33.1 (2.9)
MY ^c^	mL/g COD fed	320 (0.01)	270 (0.01)	260 (0.01)	230 (0.03)	210 (0.01)	170 (0.01)	190 (0.00)	30 (0.01)
H_2_S content	ppmv	9233 (681)	19,500 (436)	30,000 (0)	37,000 (0)	4933 (115)	4000 (0)	19,500 (866)	15,000 (0)
HPR ^d^	mL/L·d	1.17 (0.12)	3.67 (0.26)	10.71 (0.28)	15.78 (1.32)	0.38 (0.03)	0.54 (0.04)	5.31 (0.26)	1.0 (0.1)
HY ^e^	mL/g COD fed	4 (0.0)	8 (0.001)	13 (0.001)	14 (0.003)	1.4 (0.000)	1.2 (0.000)	6.5 (0.001)	9 (0.0002)
TDS ^f^	mg S/L	65.7 (10.4)	120.6 (20.3)	152.6 (3.7)	201.1 (7.1)	63.8 (11.9)	25.3 (1.2)	73.7 (4.6)	59.5 (14.9)

^a^ Standard deviations are presented in parentheses. ^b^ Methane production rate. ^c^ Methane yield. ^d^ H_2_S production rate. ^e^ H_2_S yield. ^f^ Total dissolved sulfide.

## Supplementary Materials

The following are available online at https://www.mdpi.com/2076-2607/7/12/682/s1, Table S1: Review of previous studies on methanogenesis and sulfidogenesis under mesophilic and thermophilic conditions, Table S2: Physicochemical characteristics of inoculum and substrates.
