# Supplementary material for: Temperature Effects on Methanogenesis and Sulfidogenesis during Anaerobic Digestion of Sulfur-Rich Macroalgal Biomass in Sequencing Batch Reactors
Source: Microorganisms. 2019 Dec 11;7(12):682. doi: 10.3390/microorganisms7120682 (PMC6955875; doi:10.3390/microorganisms7120682)
Supplement: Supplementary file 1 [file microorganisms-07-00682-s001.pdf]

Table S1. Review of previous studies on methanogenesis and sulfidogenesis under mesophilic and thermophilic conditions.

Mesophilic (30–37°C)					Electron flow[a]		
No.	Substrate	COD/SO_4^{2-}	Reactor type	Temperature (°C)	Methanogenesis	Sulfidogenesis	Reference
1	Methanol	0.5	UASB[b]	35	49.8%	34.3%	Wu, Liu, Feng, Kong, Jiang and Li [4]
2	Acetate and ethanol	1	UASB	35	23.5–51.8%	30.9–71.0%	Wu, Niu, Li, Hu, Mribet, Hojo and Li [15]
3	Acetate and ethanol	1	UASB	35	48.5–70.0%	28.4–51.5%	Jing, Hu, Niu, Liu, Li and Wang [14]
4	Ethanol	2–15	UASB	30	13–69%	31–87%	Hien Hoa, Liamleam and Annachhatre [16]
5	Synthetic vinasse	2	DFSBR[c]	30	29–38%	35–37%	Godoi, Foresti and Damianovic [13]
Thermophilic (50–65°C)					Electron flow[a]		

No.	Substrate	COD/SO_4^{2-}	Reactor type	Temperature (^{o}C)	Methanogenesis	Sulfidogenesis	Reference
1	Methanol	0.5	UASB	50	81.3%	8.6%	Wu, Liu, Feng, Kong, Jiang and Li [4]
2	Methanol	0.5	UASB	55	38%	53%	Vallero, Camarero, Lettinga and Lens [17]
3	Methanol	0.5	UASB	65	N.D.	96%	
4	Formate	0.5	UASB	65	91%	9%	
5	Acetate, propionate, and butyrate	0.57	UASB	55	12%	62%	Visser, Gao and Lettinga [18]
6	Methanol	5	UASB	55	85%	13%	Vallero, Treviño, Paulo, Lettinga and Lens [11]

[a] Calculated on the basis of COD conversion.

[b] Up-flow anaerobic sludge blanket

[c] Down-flow fixed-structured bed reactor

Table S2 Physicochemical characteristics of inoculum and substrates.

Parameters	Units	Anaerobic sludge	*Ulva* substrate used at each OLRs (g COD/L·d)			
			0.25	0.4	0.75	1.0
Total COD	mg/L	27789 (72)[a]	4,997 (102)	8,046 (89)	15,101 (312)	20,991 (148)
Soluble COD	mg/L	2737 (124)	945 (67)	1695 (12)	3,094 (35)	5,239 (112)
TS	mg/L	39050 (71)	5,000 (141)	8,100 (141)	14,900 (141)	24,050 (71)
TVS	mg/L	23700 (283)	4450 (71)	7100 (141)	12900 (424)	19700 (283)
TSS	mg/L	30833 (707)	3300 (141)	2567 (236)	10500 (707)	19000 (0)
VSS	mg/L	20333 (471)	3100 (141)	4000 (0)	9000 (471)	17167 (236)
Carbon	%[b]	27.8 (0.5)	31.7 (0.1)			
Hydrogen	%	4.4 (0.1)	4.6 (0.2)			
Nitrogen	%	5.4 (0.1)	2.0 (0.1)			
Sulfur	%	1.0 (0.2)	1.8 (0.6)			
Oxygen	%	22.2 (0.6)	44.2 (0.3)			

[a] Standard deviation (in parenthesis)

[b] A dry weight basis
